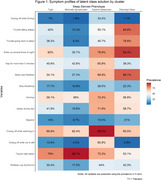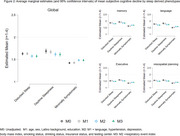# Sleep phenotypes and self‐reported cognitive decline in the Hispanic Community Health Study/Study of Latinos

**DOI:** 10.1002/alz70860_104449

**Published:** 2025-12-23

**Authors:** Kevin A Gonzalez, Rachel Membreno, Wassim Tarraf, Ariana M Stickel, Charles Decarli, Freddie Márquez, Christian Agudelo, SanJay R Patel, Linda C Gallo, Martha L Daviglus, Fernando Daniel Testai, Daniela Sotres‐Alvarez, Frank J Penedo, Barbara Junco, Hector M González, Alberto R Ramos

**Affiliations:** ^1^ University of California, San Diego, La Jolla, CA, USA; ^2^ San Diego State University, San Diego, CA, USA; ^3^ Wayne State University, Detroit, MI, USA; ^4^ University of California, Davis, Davis, CA, USA; ^5^ University of Miami Miller School of Medicine, Miami, FL, USA; ^6^ University of Pittsburg, Pittsburg, PA, USA; ^7^ University of Illinois at Chicago, Chicago, IL, USA; ^8^ UIC, Chicago, IL, USA; ^9^ University of Illinois College of Medicine, Chicago, IL, USA; ^10^ University of Miami, Coral Gables, FL, USA

## Abstract

**Background:**

Sleep problems have been associated with cognitive problems, including subjective cognitive decline (SCD), which often precedes objective cognitive changes and dementia. In recent years, phenotype‐based approaches have gained popularity in sleep research due to heterogenous presentation of sleep symptoms. Some work has found that sleep phenotypes are associated with the incidence of vascular factors, yet less is known on how these sleep phenotypes are related to SCD. In a large cohort of diverse Hispanic/Latino individuals, we examined the association between sleep phenotypes and SCD.

**Method:**

The Hispanic Community Health Study/Study of Latinos (HCHS/SOL, years 2008‐2011) is a prospective cohort study of Hispanic/Latino individual. Sleep, sociocultural, and cardiovascular information was obtained from individuals. The Study of Latinos/Investigation of Neurocognitive aging (SOL‐INCA, 2016‐2018) is an ancillary study of HCHS/SOL. Latent class analysis was performed using a gaussian mixed model on 15 sleep variables and 4 cardiovascular measures (see Figure 1) to cluster individuals into different sized sleep clusters (2 to 10). Fit statistics were obtained and used to determine optimal cluster size. We used the brief Everyday Cognition (ECog‐12) to assess SCD (at SOL‐INCA visit) in the following subdomains: memory, executive, visuospatial planning, language, and a global composite (mean of all domains). SCD was defined by a score of 1‐4 with larger values meaning more SCD. We used survey weighted linear regression models to assess the relationship between sleep phenotypes and SCD. A full list of covariates can be found in Figure 2.

**Result:**

*N* = 5,551 individuals were included in the analysis. The three‐class solution was the best fit. The prevalence of sleep symptoms can be found in Figure 1. Results from linear models can be found in Figure 2. Individuals in the disturbed sleep group (*N* = 1,886) and the daytime sleepiness group (*N* = 943) had more SCD (higher scores) across all domains compared to those in the minimally symptomatic group (*N* = 2,722). Associations remained after covariate adjustments.

**Conclusion:**

Both disturbed sleep and daytime sleepiness were associated with more SCD compared to the minimally symptomatic group. Our findings show that sleep phenotype approaches could be potentially useful for understanding cognitive health, particularly in Hispanic/Latino populations.